# Effects of V4c-ICL Implantation on Myopic Patients' Vision-Related Daily Activities

**DOI:** 10.1155/2016/5717932

**Published:** 2016-11-14

**Authors:** Taixiang Liu, Shaorong Linghu, Le Pan, Rong Shi

**Affiliations:** Guizhou Ophthalmic Hospital, The Affiliated Hospital of Zunyi Medical College, Zunyi 563003, China

## Abstract

The new type implantable Collamer lens with a central hole (V4c-ICL) is widely used to treat myopia. However, halos occur in some patients after surgery. The aim is to evaluate the effect of V4c-ICL implantation on vision-related daily activities. This retrospective study included 42 patients. Uncorrected visual acuity (UCVA), best corrected visual acuity (BCVA), intraocular pressure (IOP), endothelial cell density (ECD), and vault were recorded and vision-related daily activities were evaluated at 3 months after operation. The average spherical equivalent was −0.12 ± 0.33 D at 3 months after operation. UCVA equal to or better than preoperative BCVA occurred in 98% of eyes. The average BCVA at 3 months after operation was −0.03 ± 0.07 LogMAR, which was significantly better than preoperative BCVA (0.08 ± 0.10 LogMAR) (*P* = 0.029). Apart from one patient (2.4%) who had difficulty reading computer screens, all patients had satisfactory or very satisfactory results. During the early postoperation, halos occurred in 23 patients (54.8%). However there were no significant differences in the scores of visual functions between patients with and without halos (*P* > 0.05). Patients were very satisfied with their vision-related daily activities at 3 months after operation. The central hole of V4c-ICL does not affect patients' vision-related daily activities.

## 1. Introduction

Posterior chamber phakic intraocular lens (pIOL) implantation is generally accepted as an effective and reversible treatment method for high myopia with preservation of lens-regulating capability. In the ophthalmology clinic, the implantable Collamer lens (ICL, STAAR Surgical, Nidau, Switzerland) is the main posterior chamber pIOLs; it is designed to be placed between the iris and the anterior surface of the lens and is fixed to the ciliary sulcus by four haptics, thus preventing its contact with the lens [1].

Although pIOL implantation has a good clinical efficacy in the correction of high myopia, some short-term and long-term complications have been reported. Cataract is the most common complication after ICL implantation. This complication may be caused by direct contact of the ICL with the lens due to a low vault or insufficient circulation of the aqueous humor. The incidence of cataract after ICL implantation ranges between 0.61% and 2.7%, depending on different follow-up periods in different studies [2–4].

High intraocular pressure is a major concern after ICL implantation, particularly for early ICL models [5–8]. To avoid postoperative elevation of intraocular pressure, peripheral iridectomy should be performed before or during ICL implantation. However, iridectomy may lead to pain and intraoperative bleeding and increase difficulty performing the operation.

To avoid peripheral iridectomy, the V4c-ICL model with a 0.36 mm central hole has been designed based on the V4-ICL model. Because the central hole of the V4c-ICL facilitates the outflow of the aqueous humor, peripheral iridectomy is not required. In addition, compared with the traditional V4-ICL model, the V4c-ICL model exhibits similar low-, middle-, and high-frequency contrast sensitivity and higher-order aberrations under the conditions of various pupil sizes, and the subjective symptoms such as glare or halo were also essentially equivalent, thus suggesting that the V4c-ICL model has good safety and efficacy [9, 10].

Although studies have investigated the effect of V4c-ICL implantation on patients' visual function status, to our knowledge, the effect of V4c-ICL implantation on visual activity in the complex environment of the patients' daily lives has not been studied. Furthermore, some patients complain of halos after V4c-ICL implantation. It remains to be determined whether this subjective symptom can affect the patient's daily visual activity. Therefore, in this study, we conducted a questionnaire to investigate the effects of V4c-ICL implantation on myopic patients' vision-related daily activities.

## 2. Material and Methods

### 2.1. Patients

This retrospective study collected data from 42 consecutive patients (82 eyes) with complete clinical data who underwent V4C-ICL implantation at the Affiliated Hospital of Zunyi Medical College (Zunyi, China) between November 2014 and November 2015. All patients were followed up for more than 3 months (range, 3 to 6 months; mean, 4.62 ± 1.23 months). Fourteen patients were male and 28 patients were female. In 2 cases, V4c-ICL implantation was performed in 1 eye. In 40 cases, V4c-ICL implantation was performed in both eyes.

The patients' average age was 24.04 ± 4.75 years (range, 18–35 years). The average preoperative sphere power was −10.21 ± 3.02 diopter (D) (range, −4.0 D to −15.0 D), and the average cylinder power was −2.48 ± 0.91 D (range, −1.25 D to −4.5 D). The average spherical equivalent (SE) was −11.55 ± 3.52 D (range, −5.75 D to −16.25 D). The preoperative and postoperative uncorrected visual acuity (UCVA) and best corrected visual acuity (BCVA) of the patients were recorded using the decimal method and converted into the LogMAR (logarithm of the minimal angle of resolution) equivalence.

Preoperative and postoperative intraocular pressure and ECD were measured. UBM was used to detect the central and peripheral vault at 3 months after operation. Three months after operation, all patients were asked by the same doctor to evaluate the visual function. The questionnaire was designed based on the visual function evaluation questionnaire used by the Corneal Diseases and Excimer Laser Research Unit, University of Dundee, Scotland [11], with slight modifications. Since some patients complained of halos after V4c-ICL implantation, we also investigated the effect on the visual functions of the presence of a halo in patients during the follow-up. This study was performed according to the Declaration of Helsinki, and all patients gave their informed consent after a comprehensive explanation of the possible risk of V4c-ICL implantation.

Inclusion criteria were BCVA of 0.5 or above and refractive stability for more than 2 years. Exclusion criteria were age < 18 years; anterior chamber depth < 2.8 mm, ECD < 2000/mm^2^; corneal diseases; and a history of eye diseases affecting visual function such as glaucoma, cataract, retinal diseases, uveitis, and ocular trauma.

### 2.2. Surgical Procedure

The size of the V4c-ICL was determined by the horizontal white-to-white corneal diameter and anterior chamber depth of the patients. The power of V4c-ICL was calculated using the software provided by the manufacturer (STAAR Surgical). Fifteen minutes before operation, compound tropicamide eye drops were applied to dilate the pupils, followed by topical anesthesia with 0.4% oxybuprocaine hydrochloride. For patients implanted with V4c-ICL for astigmatism, the astigmatic axis was marked using a slit lamp. The main incision site was created at the position of 135° and an auxiliary incision site was made at the position of 45°.

After introduction of viscoelastic materials to maintain the anterior chamber, the V4c-ICL was slowly pushed into the anterior chamber using an injector cartridge. Then, the haptics of the ICL was enclaved into the anterior chamber via the main and auxiliary incision sites using the manipulator. After the axis was adjusted, the remaining viscoelastic materials were replaced. The use of miotics was dependent on the pupil size. Anti-inflammatory treatment was applied after the operation.

### 2.3. Statistical Analysis

Numerical data are presented as mean ± SD. Analyses were performed using SPSS v17.0 software. Repeated analysis of variance was used to analyze the differences in intraocular pressure as well as ECD at different timepoints before and after the operation. Independent Student's *t*-test was used to analyze the differences in the central vault and peripheral vault and the score of visual analog scale between different groups. Probability values less than 0.05 were considered statistically significant.

## 3. Results

### 3.1. Refractive Status and Vision

The average preoperative spherical equivalent was −11.55 ± 3.52 D (range, −5.75 D to −16.25 D). The average spherical equivalent was −0.12 ± 0.33 D (range, −1.00 to −0.50 D) and the residual astigmatism was −0.18 ± 0.32 D (range, 0.25 to −1.25 D) at 3 months after patients operations, excluding those patients with preexisting astigmatism who did not receive the implanted astigmatic V4c-ICL model and required preservation of astigmatic power.

The average preoperative UCVA was 1.47 ± 0.23 LogMAR (range, 1.15 to 2.0). The average UCVA was −0.03 ± 0.08 LogMAR (range, −0.18 to 0.30) at 3 months after operation. The uncorrected visual acuity had increased significantly at 3 months after operation compared with before operation (*p* < 0.001). At 3 months after operation, UCVA in 80 eyes (98%) was equal to or better than preoperative BCVA. The efficacy index (= postoperative UCVA/preoperative BCVA) was 1.27.

The average BCVA at 3 months after operation was −0.03 ± 0.07 LogMAR (range, −0.18 to 0.22), which was significantly better than preoperative BCVA (average, 0.08 ± 0.10 LogMAR; range, −0.08 to 0.40) (*p* = 0.029). No patients had postoperative BCVA worse than preoperative values. Thirty eyes (36%) had postoperative BCVA equal to preoperative values. Postoperative BCVA increased by one line in 26 eyes (32%). Postoperative BCVA increased by two lines in 18 eyes (22%). Postoperative BCVA increased by three lines in 8 eyes (10%). The safety index (= preoperative BCVA/postoperative BCVA) was 1.28.

### 3.2. Intraocular Pressure

**Figure 1:**
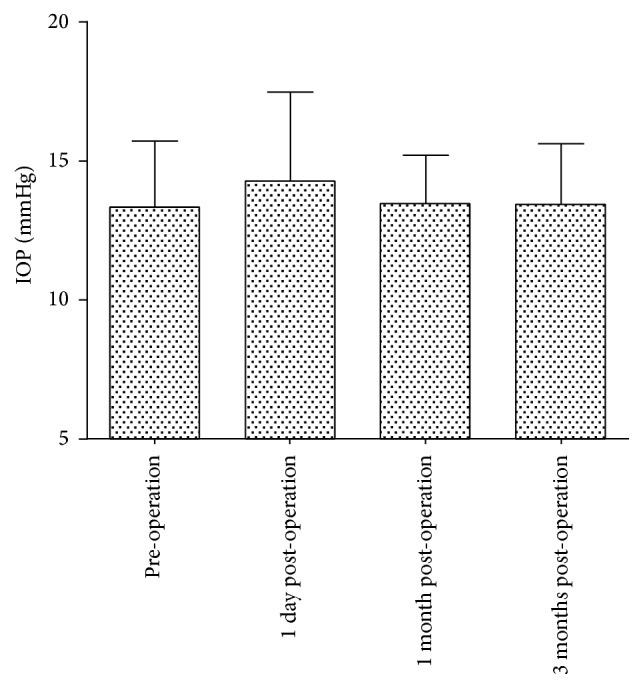
The IOP before and at 1 day, 1 month, and 3 months after V4c-ICL implantation.

The average preoperative intraocular pressure (IOP) was 13.35 ± 2.34 mmHg (range, 9.7 to 18.0 mmHg). One day after operation, the IOP was slightly increased (average, 14.26 ± 3.20 mmHg; range, 10.0 to 25.0 mmHg). Only one eye had an IOP > 21 mmHg, and the IOP returned to the normal level (14 mmHg) without any special treatment at the second day after surgery. At 1 month after operation, the IOP was stable (average, 13.46 ± 1.74 mmHg; range, 10.0 to 17.2 mmHg). At 3 months after operation, the average IOP was 13.42 ± 2.19 mmHg (range, 10.0 to 17.0 mmHg), which was not significantly different from that before (and at 1 month) and after operation (Figure 1).

### 3.3. Corneal Endothelial Cell Density

**Figure 2:**
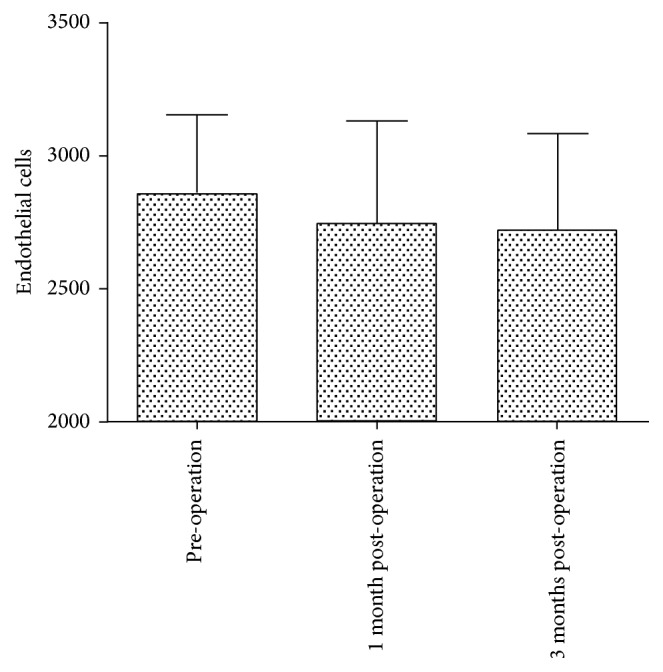
The ECD before and at 1 month and 3 months after V4c-ICL implantation.

ECD was 2857.76 ± 295.60 before operation, 2745.59 ±  384.11 at 1 month after operation, and 2719.30 ± 363.02 at 3 months after operation. Compared with the preoperative value, ECD at 1 month and 3 months after operation decreased by 3.92% (*p* = 0.144) and 4.83% (*p* = 0.065), respectively (Figure 2).

### 3.4. Vault

**Figure 3:**
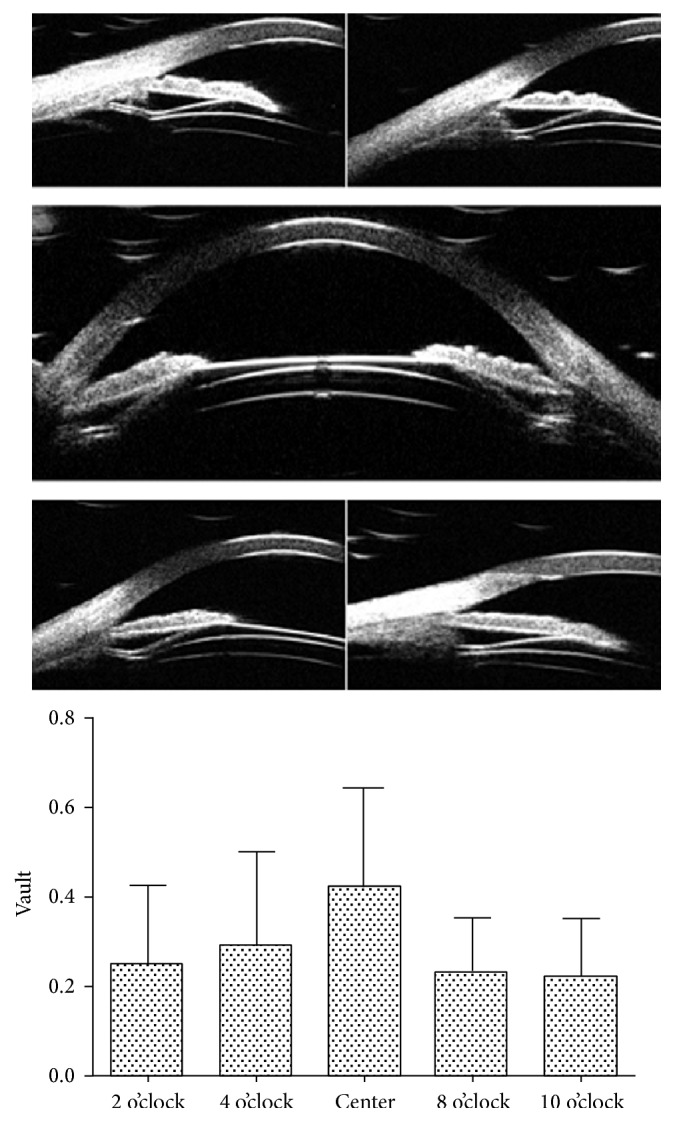
The peripheral vault and central vault at 3 months after V4c-ICL implantation.

We investigated the peripheral vault (the perpendicular line between the end of suspensory ligament and the ICL) and the central vault (the perpendicular line between the surface of the anterior lens capsule and the ICL surface) at 3 months after ICL implantation (Figure 3). The peripheral vault was 0.25 ± 0.17 mm (range, 0.03 to 0.92 mm) at 2 o'clock, 0.29 ± 0.21 mm (range, 0.05 to 0.92 mm) at 4 o'clock, 0.23 ± 0.12 mm (range, 0.06 to 0.64 mm) at 8 o'clock, and 0.22 ± 0.13 mm (range, 0.01 to 0.80 mm) at 10 o'clock. The central vault was 0.42 ± 0.22 mm (range, 0.13 to 0.90 mm), which was significantly higher than the peripheral vaults (*p* = 0.000, 0.003, 0.000, and 0.000, resp.).

In one eye, the peripheral vault at 2 o'clock and 4 o'clock was as high as 0.92 mm, and the peripheral vault at 8 o'clock and 10 o'clock was 0.46 mm and 0.44 mm, respectively. The central vault was 0.9 mm. The high vault value may be due to ICL tilt after implantation. During the 6-month follow-up, the patients had visual acuity of −0.08, normal IOP (11–16 mmHg), no endothelial damage, and normal daily visual activities. Therefore, we did not exchange or reposition the tilt ICL.

### 3.5. Visual Function

The patients were required to fill out a questionnaire about visual functions at 3 months after operation. Complete questionnaires were returned by 42 patients and all of these patients answered the questionnaire satisfactorily.

In Table 1, items 1-2 evaluated near vision, items 3–5 evaluated far vision, item 6 was for night vision, and items 7–11 were for middle-distance vision. We evaluated the daily activities associated with near vision, far vision, and middle-distance vision. Apart from one patient who had a difficulty reading computer screens, all patients had satisfactory or very satisfactory results. We also investigated whether the patients had a halo after operation. During the early postoperative follow-up period, halos occurred in 23 patients (54.8%). With time, halos gradually disappeared at 3 months after operation without any treatments. The patients were categorized into two groups: patients with halos and patients without halos. The average age in patients with halos was 25.5 ± 4.3 years, which was not significantly different from those without halos (23.2 ± 5.0 years) (*p* = 0.702). There were no significant differences in the scores of visual functions between the two groups (*p* > 0.05, Table 2).

## 4. Discussion

Posterior chamber pIOL implantation is an effective refractive surgery that has been widely accepted. However, elevated intraocular pressure is a common postoperative complication after pIOL implantation, even in patients with preoperative or intraoperative iridectomy [5].

To simplify the ICL implantation procedure and improve postoperative aqueous circulation, V4c-ICL with a 0.36 mm central hole was developed. Clinical studies have shown that V4c-ICL implantation is effective in the treatment of moderate and high myopia [9, 12, 13].

In the present study, we found that (after V4c-ICL implantation) UCVA was equal to or better than preoperative BCVA in 80 eyes (98%) with high myopia. No patients had postoperative BCVA worse than preoperative BCVA. Almost all eyes had BCVA equal to or better than preoperative BCVA by one line or more. The postoperative UCVA and BCVA were −0.03 ± 0.08 LogMAR and −0.03 ± 0.07 LogMAR, respectively. The efficacy index and safety index were 1.27 and 1.28, respectively, which were consistent with previous studies [9, 12–14]. These findings suggest that V4c-ICL is a safe and effective treatment for high myopia, and it significantly simplifies the ICL implantation procedure. During postoperative follow-up periods, intraocular pressure was stable and only slightly increased on postoperative day 1. Due to the presence of the central hole, V4c-ICL implantation avoids preoperative or intraoperative peripheral iridectomy, thus reducing IOP elevation caused by surgical stimulation, depigmentation, and pupillary block. This may explain the similar IOP before and after V4C-ICL implantation.

In the present study, we found that the ECD at 1 month and 3 months after operation were reduced by 3.92% and 4.83%, respectively. Compared with the preoperative value, postoperative ECD was not significantly decreased, which was consistent with the results reported by Shimizu et al. [13]. Although we found that V4c-ICL implantation did not produce a short-term effect on ECD, it remains to be determined whether aqueous outflow through the central hole of the V4c-ICL has a long-term effect on ECD. Further studies with long-term follow-ups and large sample sizes should be performed.

Vault is one of the most important indices for evaluating the postoperative effect of the ICL implantation. In the present study, we performed UBM to observe the relative position between the V4c-ICL and the lens and found that no V4c-ICL was in direct contact with the lens. The peripheral vault at each measured site was significantly lower than the central vault. This may occur because the center of the V4c-ICL is designed to be thinner than the periphery.

Evaluation of visual quality is a comprehensive method to evaluate the effect of refractive surgery and has been widely used. It has been reported that there is no significant difference in visual outcome between V4c-ICL and V4-ICL implantation [9, 10]. However, these studies only investigated the effect of V4c-ICL and C4-ICL implantation on contrast sensitivity and higher-order aberration. There were no reports about whether it affects the daily visual functions after implantation of V4c-ICL. In the present study, we investigated the effect of V4c-ICL implantation on vision-related daily activities during follow-up. We found that apart from one patient (2.4%) who had a difficulty in reading computer screen, all patients had satisfactory or very satisfactory results. In addition, we found that there were tilt ICL in one eye of another patient. During the 6-month follow-up, the patients with tilt ICL had visual acuity of −0.08, normal IOP (11–16 mmHg), no endothelial damage, and normal daily visual activities. In our short-term follow-up, even the tilt of the V4c-ICL could not bring a serious impact on the patient. Of course it was necessary to observe the patient for a long term. During the early postoperative follow-up period, halos occurred in more than 50% (54.8%, 23 patients) of patients. With time, halos gradually disappeared after operation without treatment. There were no significant differences in the scores of visual functions between patients with or without halos. These findings suggest that halos do not affect postoperative vision-related daily activities.

In conclusion, we investigated the vision-related daily activities at 3 months after V4c-ICL implantation. We found that the patients were satisfied with their vision-related daily life activities, and the presence of central hole of V4c-ICL did not affect vision-associated daily activities. Postoperative intraocular pressure was stable, and no vision-associated complications were found during the follow-up period. However, because V4c-ICL implantation alters aqueous circulation, it remains to be determined whether V4c-ICL implantation produces long-term effect on intraocular pressure and ECD.

## Figures and Tables

**Table 1:** Visual functions of patients after V4c-ICL implantation (*n* = 42).

Visual functions	Mean score ± SD	Very positive% (number of patients)	Positive% (number of patients)	Negative% (number of patients)	Very negative% (number of patients)
Reading in daylight	8.3 ± 0.9	83.3 (35)	16.7 (7)	0 (0)	0 (0)
Reading in artificial light	8.0 ± 1.1	71.4 (30)	28.6 (12)	0 (0)	0 (0)
Watching TV	8.0 ± 1.1	71.4 (30)	28.6 (2)	0 (0)	0 (0)
Watching movie at cinema	8.0 ± 1.1	71.4 (30)	28.6 (12)	0 (0)	0 (0)
Driving in daylight	8.3 ± 1.0	81.0 (34)	19.0 (8)	0 (0)	0 (0)
Driving at night	7.8 ± 1.2	61.9 (26)	38.1 (16)	0 (0)	0 (0)
Reading computer screen	7.5 ± 1.4	52.4 (22)	45.2 (19)	2.4 (1)	0 (0)
Playing sports	8.2 ± 1.0	78.6 (33)	21.4 (9)	0 (0)	0 (0)
Swimming	8.1 ± 1.1	73.8 (31)	26.2 (11)	0 (0)	0 (0)
Shaving/makeup	8.0 ± 1.1	71.4 (30)	28.6 (12)	0 (0)	0 (0)
Shopping	8.3 ± 1.0	81.0 (34)	19.0 (8)	0 (0)	0 (0)

**Table 2:** Visual functions after 4c-IC implantation in patients with (+) or without (–) halo.

Visual functions	Halo		
	−	+	*p*
	*n* = 19	*n* = 23	
Reading in daylight	8.8 ± 0.0	8.4 ± 1.0	0.165
Reading in artificial light	8.3 ± 1.0	8.2 ± 1.1	0.780
Watching TV	7.8 ± 1.3	8.2 ± 1.1	0.497
Watching movie at cinema	8.3 ± 1.0	7.6 ± 1.3	0.152
Driving in daylight	8.8 ± 0.0	8.2 ± 1.1	0.082
Driving at night	8.1 ± 1.2	8.0 ± 1.2	0.859
Reading computer screen	7.8 ± 1.3	7.0 ± 1.6	0.178
Playing sports	8.8 ± 0.0	8.2 ± 1.1	0.082
Swimming	8.5 ± 0.8	8.0 ± 1.2	0.194
Shaving/makeup	8.3 ± 1.0	8.0 ± 1.2	0.500
Shopping	8.8 ± 0.0	8.4 ± 1.0	0.082
